# Bioengineering of *Bordetella pertussis* Adenylate Cyclase Toxin for Vaccine Development and Other Biotechnological Purposes

**DOI:** 10.3390/toxins13020083

**Published:** 2021-01-22

**Authors:** Daniel Ladant

**Affiliations:** Biochemistry of Macromolecular Interactions Unit, Structural Biology & Chemistry Department, Institut Pasteur CNRS UMR, CEDEX 15, 3528 Paris, France; daniel.ladant@pasteur.fr

**Keywords:** *Bordetella pertussis*, adenylate cyclase, recombinant toxin, antigen delivery, recombinant vaccine, biological screening, protein–protein interactions, two-hybrid

## Abstract

The adenylate cyclase toxin, CyaA, is one of the key virulent factors produced by *Bordetella pertussis*, the causative agent of whooping cough. This toxin primarily targets innate immunity to facilitate bacterial colonization of the respiratory tract. CyaA exhibits several remarkable characteristics that have been exploited for various applications in vaccinology and other biotechnological purposes. CyaA has been engineered as a potent vaccine vehicle to deliver antigens into antigen-presenting cells, while the adenylate cyclase catalytic domain has been used to design a robust genetic assay for monitoring protein–protein interactions in bacteria. These two biotechnological applications are briefly summarized in this chapter.

## 1. Introduction

*Bordetella pertussis* is a strictly human pathogen that is the causative agent of whooping cough, or pertussis, a respiratory infection that still kills over a 100,000 children annually, mainly in developing countries [1]. *B. pertussis* is a Gram-negative bacterium that colonizes the respiratory tract. It produces a number of adhesins and virulence factors, including the filamentous hemagglutinin (FHA), pertussis toxin (PTX), and the adenylate cyclase toxin (CyaA), which are all essential for pathogenesis [2,3]. CyaA plays a critical role in the early stages of bacterial respiratory tract colonization. This toxin primarily targets macrophages and neutrophils, and helps bacteria to fight against the primary innate immune responses [2,3]. CyaA invades eukaryotic target cells and delivers its catalytic domain into the cell cytosol, where it is activated upon binding to endogenous calmodulin (CaM), which stimulates its enzymatic activity to produce a massive amount of cAMP [4,5,6,7]. The hyperphysiological cAMP levels drastically impair the target immune cells’ bactericidal capacity such as chemotaxis, superoxide anion generation by peripheral blood monocytes, neutrophil, and macrophage phagocytosis, and they can trigger macrophage apoptosis.

**Figure 1:**
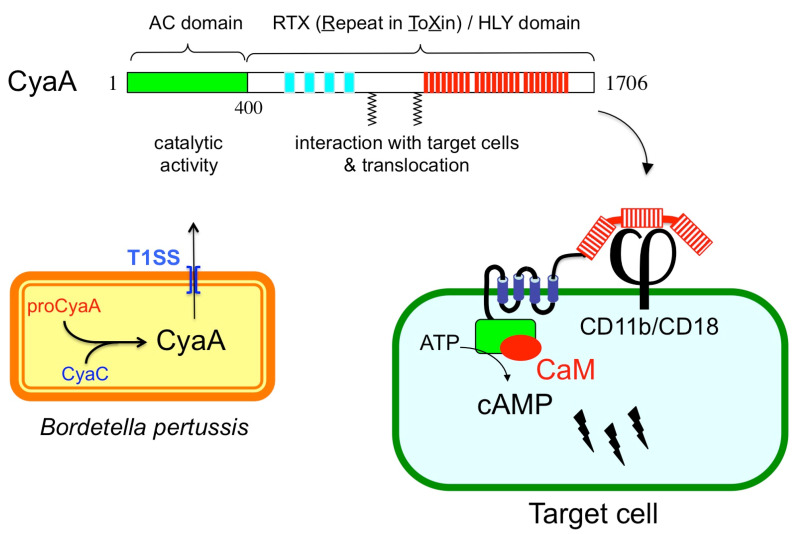
Schematic representation of CyaA structural organization and biogenesis. Upper part: Structural organization of the CyaA polypeptide with its different subdomains. Lower part left: in *B. pertussis,* CyaA is synthesized as an inactive precursor proCyaA that is activated by the CyaC acyltransferase and then secreted across the bacterial envelope by a dedicated type I secretion system (T1SS); Lower part right: model for CyaA invasion of eukaryotic cells. After binding to CD11b/CD18 receptor (j), CyaA inserts its hydrophobic segments into the membrane and delivers its catalytic domain into the cytosol where it is activated by calmodulin (CaM) to overproduce cAMP.

CyaA is a member of a large family of bacterial cytolysins known as RTX (Repeat-in-ToXin) toxins [8,9]. CyaA is a 1706-residue long bifunctional protein (Figure 1) made of a CaM-activated catalytic domain (AC) located in the ~400 amino-proximal residues and appended to a 1300-residue long carboxy-terminal moiety that displays all the characteristics of RTX cytolysins [9]. This C-terminal region is indeed endowed with hemolytic activity. The RTX cytolysins are pore-forming toxins that contain characteristic tandem repetitions of nonapeptidic, “Repeat-in-ToXin” (RTX) motifs that constitute specific calcium-binding sites. CyaA contains ~40 copies of RTX motifs (residues 913–1613), and calcium is essential for CyaA cytotoxicity. CyaA is synthesized as an inactive precursor proCyaA that is converted into an active toxin upon selective acylation of two lysine residues, K860 and K983, a modification that is carried out by a dedicated acyltransferase, CyaC. The acylated CyaA polypeptide is then secreted across the bacterial envelope by a specifc type I secretion machinery (T1SS) made of three components encoded by the *cyaB*, *cyaD*, and *cyaE* genes, which form an operon with the *cyaA* gene [3,9]. Although CyaA can intoxicate a variety of cell types by a process that is independent of receptor-mediated endocytosis, it binds in a calcium-dependent manner to the CD11b/CD18 integrin [10]. This receptor is expressed by a subset of leukocytes including neutrophils, macrophages, and dendritic cells (DC), and consequently these cells are the natural in vivo targets of CyaA during *B. pertussis* infection [4,7]. After binding to the CD11b/CD18 receptor, the hydrophobic region (aa 500 to 750) of CyaA is inserted into the plasma membrane to firmly anchor the toxin to the target cells. Finally, the catalytic domain is directly delivered across the membrane into the cytosol, where it binds CaM and produces cAMP [4,7,11,12]. This direct delivery of the CyaA catalytic domain across the plasma membrane of the target cells is rather unique among bacterial toxins that usually exploit diverse routes of endocytosis to enter eukaryotic cells. One consequence of this original entry pathway is that CyaA can trigger a very rapid increase of intracellular cAMP, which is mainly localized in the vicinity of the plasma membrane [13]. This spatio-temporal compartmentalization of the cAMP signaling likely contributes to the specific mode of action of CyaA on different target cells [14,15,16]. Currently, the precise molecular mechanisms by which the AC domain can pass across the hydrophobic barrier of the lipid bilayer cells remain the subject of active investigations. Fundamental aspects of the biogenesis of CyaA, including the biophysics of secretion, calcium and acylation-dependent folding, and toxin translocation across membranes have been further discussed in several recent articles and reviews [4,7,12]. The remaining chapter will deal with the biotechnological exploitation of the original characteristics of CyaA in vaccinology and biotechnology.

## 2. Engineering CyaA as a Molecular Trojan Horse to Deliver Antigens into Antigen-Presenting Cells

**Figure 2:**
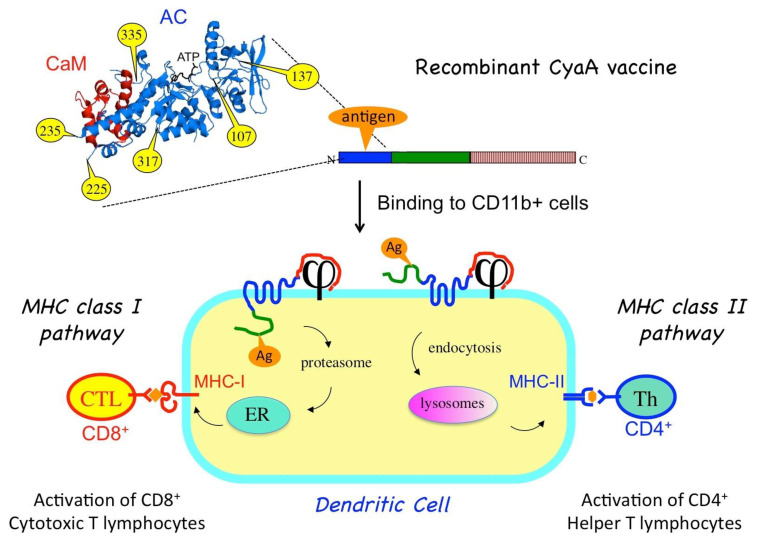
Recombinant CyaA for antigen delivery into dendritic cells (DCs). An antigen of interest (orange) is genetically inserted into one of the permissive sites (yellow) located within the CyaA catalytic domain. When injected into an animal, the purified CyaA-antigen fusion binds to the CD11b/CD18 receptor (j) at the surface of the dendritic cells. Upon translocation of the AC domain across the plasma membrane, the antigen is processed by proteasome and enters the MHC-I presentation pathway (via the endoplasmic reticulum, ER) leading to the activation of cytotoxic CD8+ T-cells. Alternatively, the recombinant CyaA is endocytosed, and the grafted antigen enters the endosomal pathway for MHC-II presentation, leading to activation of specific CD4+ helper T-cells.

The CyaA toxin presents two key characteristics that are particularly favorable to designing a molecular Trojan horse in order to deliver antigens into antigen-presenting cells:

(1) CyaA binds with high affinity to the CD11b/CD18 integrin [10]. This integrin is expressed mainly on innate immune cells such as macrophages and neutrophils as well as by a subpopulation of dendritic cells (DCs). Indeed, CyaA was shown to specifically target the CD11b^+^ DCs subset in vivo [17]. The DCs are the main professional antigen-presenting cells (APCs), the function of which is to process antigens to present them on their cell surfaces to stimulate the T cells of the adaptive immune system [18]. Given the central role of these cells in the initiation of the adaptive immune response, efficient targeting of antigens to DCs represents a crucial component of modern vaccination strategy. The natural tropism of CyaA for the CD11b^+^ DC subset turned out to be of the utmost advantage for a potential vaccine vehicle.
(2) CyaA can tolerate genetic insertion of polypeptide fragments of a relatively large size (up to a few hundred residues) into its N-terminal catalytic domain without impairing its ability to translocate across the plasma membrane of eukaryotic cells [19]. Therefore, recombinant CyaA vaccines can be easily designed by genetically grafting antigens of interest onto the so-called permissive sites within the catalytic domain of the toxin [20] (Figure 2). To avoid potential toxicity of the recombinant CyaAs due to the cAMP-synthetizing capacity, the enzymatic activity is generally ablated by specific mutations within the catalytic site [19].

The recombinant toxins can be produced in large amount in *Escherichia coli* and purified to homogeneity using robust procedures to yield vaccine molecules that upon injection into an animal or patient can be efficiently targeted in vivo to CD11b^+^ DCs. In these professional antigen-presenting cells, the grafted antigens are then processed and presented to trigger specific cellular immune responses [7].

The capacity of CyaA to deliver its AC domain to the cytosol is particularly appropriate for inducing specific cytotoxic T lymphocytes (CTL) or CD8+ T cells. The CD8+ T-cells recognize epitopes that are presented by the molecules of the major histocompatibility complex (MHC) of class I (MHC-I). These epitopes are mainly derived from cytosolic proteins after processing by the proteasome into peptides that are transported (by the “TAP” transporters) into the endoplasmic reticulum, where they associate with nascent MHC-I molecules. The epitope/MHC-I complexes are then routed to the cell surface where they can be recognized by T cells via their specific T cell receptor (TcR). Engagement of the CD8+ T-cells results in specific lysis of the cells presenting the epitopes. CD8+ T cells are thus essential for eliminating cells infected by virus or other intracellular pathogens as well as to kill tumor cells that express abnormal proteins (commonly referred to as tumor-associated antigens). Induction of potent cytotoxic cell responses is therefore a major goal of all successful vaccine strategies [18] and CyaA is one of the first non-replicating proteins shown to be able to do so in animal models [21]. In vitro experiments further demonstrated that T-CD8+ epitopes (actually, a model epitope derived from ovalbumin) that have been grafted onto recombinant CyaAs are processed and presented according to the so-called classical cytosolic MHC-I presentation pathway, depending upon proteasome activity, active peptide-specific transporter TAP, and neosynthesis of MHC-I molecules [22].

Importantly, antigens grafted in the catalytic domain of CyaA can also be routed to the MHC class II presentation pathway [23]. MHC class II molecules bind to peptides that are derived from proteins degraded in the endocytic pathway. MHC class II molecules loaded with CD4 epitopes are then transported to the cell surface where the peptide/MHC-II complexes are recognized by the T cell receptor of specific CD4+ helper T lymphocytes. When activated, the helper T cells produce a variety of specific cytokines and chemokines that are critical growth factors for B lymphocytes that produce antibodies as well as for CD8+ T cells and antigen-presenting cells. Schlecht et al. [24] showed that CyaA can simultaneously deliver epitopes into both the MHC I- and II-restricted presentation pathways, likely via two distinct routes. As illustrated in Figure 2, direct membrane translocation of the recombinant CyaA will feed the MHC-I presentation pathway while after endocytosis, the CyaA–Ag will be degraded/processed in the endosomal/lysosomal compartment to reach the MHC II presentation path [25,26].

In addition to the induction of CD4 and CD8 T cell responses, CyaA is also able to trigger potent humoral immune responses against the grafted antigens as exemplified by studies done with the Tat (transactivator of transcription) protein, a critical virulence factor from the human immunodeficiency virus. A CyaA-Tat protein was shown to elicit strong Th1-polarized CD4+ responses and IFN-γ producing CD8+ T-cells, as well as potent anti-Tat antibody responses capable of neutralizing in vitro the transactivating activity of the native Tat protein [27]. These results were obtained in mice in the absence of any adjuvant as well as in a non-human primate model (African green monkeys) [28].

Another important property was uncovered by Dadaglio et al., who showed that CyaA can not only target DCs but is also able to induce the maturation of these cells via a TLR4/TRIF pathway [29]. This capacity, which is independent of the cAMP-synthetizing activity, likely explains the strong potency of CyaA in inducing specific T cell responses.

All these studies thus firmly established that CyaA represents an attractive antigen vehicle for vaccinal purposes.

A variety of recombinant CyaA proteins carrying various viral, bacterial, or tumoral epitopes or antigenic fragments have been produced and shown to stimulate strong T cell responses against the grafted epitopes when injected into animals [21,30,31,32,33]. Importantly, in a mice model of infection, a recombinant CyaA carrying a single epitope from the lymphocytic choriomeningitis virus (LCMV) was able to protect immunized animals against viral infections with lethal doses of the virus [30]. Other experiments demonstrated the efficacy of CyaA to induce strong T cell responses in mice against key antigens from *Mycobacterium tuberculosis* [34,35].

Recombinant CyaA proteins carrying epitopes derived from tumor-associated antigens (including the model antigen ovalbumin, see above), were shown to induce potent epitope-specific CTL responses in mice and conferred a protective immunity characterized by significantly delaying the growth of tumor cells, resulting in a prolonged survival of the immunized animal, compared to control animals that received a placebo or unrelated CyaA protein [31]. Furthermore, the CyaA vaccines were also effective in a therapeutic mode, that is, when administered to the animals after the tumor graft, a key feature for potential application to human.

The most advanced studies of antitumoral immunity were obtained in a mouse model of human papillomavirus (HPV)-induced tumors. HPV-induced cervical carcinoma is one of the most prevalent gynecologic cancers in the world and the focus of major vaccinal efforts [36]. The currently marketed prophylactic HPV vaccines must be administered prior to exposure to HPV, and cannot treat HPV infection or HPV-associated diseases such as cancer. Development of therapeutic HPV vaccines remains a high priority [37]. Most of them target two key HPV oncoproteins (E6 and E7), which are essential for driving the infected cells toward oncogenesis [36]. Recombinant CyaAs carrying the E7 oncoprotein (either full-length or fragments) from the HPV16 subtype (the most prevalent) were produced and shown to induce potent Th1 and CTL responses in mice. When administrated intradermally in the absence of any adjuvant, the recombinant CyaA-HpvE7 proteins were able to completely eradicate pre-established E7-expressing tumors in all tested animals, thereby demonstrating the potency of the vaccine [33,38].

From this “proof of concept”, the biotech company Genticel (now merged with Genkyotex) set up clinical trials to evaluate the efficacy of CyaA-based vaccine in humans for immunotherapy of HPV-induced tumors [39,40]. In a phase I clinical trial (EudraCT No. 2010-018629-21), a bivalent vaccine, GTL001, constituted of two different detoxified CyaAs carrying the E7 antigen from HPV-16 and HPV-18 (the two most prevalent subtypes associated with cervical cancer), was evaluated in women infected with HPV16 or 18 but with normal cytology. The vaccine and placebo were administered by intradermal injections followed by topical applications of imiquimod (Aldara^TM^) a synthetic imidazoquinolone that stimulates NF-kB (via TLR 7 and TLR 8) to induce pro-inflammatory cytokines and Th1 responses. The phase I trial showed a good safety profile and also suggested that GTL001/imiquimod could facilitate HPV clearance [39,40]. Yet, a larger phase II study aimed at evaluating vaccine efficacy failed to indicate any significant difference in viral clearance in treated patients compared to the placebo group over a two-year period, leading to the termination of the GTL001 vaccine development program. These results highlight how much cancer immunotherapy remains challenging. A variety of therapeutic vaccine candidates for HPV-related malignancies are currently being tested but with limited success until now [37]. More successful strategies will likely arise from combining these different approaches, as well as from co-administration of immunomodulatory agents to modulate the tumor microenvironment and immune checkpoint inhibitors [37,41,42]. For example, the combination of CyaA-HPV-E7 with CpGs (TLR9 ligand) and cyclophosphamide (to suppress regulatory T cells) was shown to be effective in eliminating large E7-expressing murine tumors in mice models [38]. It may also be valuable to engineer the CyaA vaccine to improve its efficacy in antigen delivery and enhance antigen processing. Finally, a better understanding of the physico-chemistry of the CyaA vector, in particular regarding the cell invasion process and mechanisms of DC stimulation, will help in the design of more efficient vaccines.

## 3. Application of CyaA for Biotechnological Screening of Protein–Protein Interactions: A Bacterial Adenylate Cyclase-Based Two-Hybrid (BACTH) Assay

The catalytic domain (AC) of CyaA corresponds to the first 370 residues of the toxin. It binds with high affinity (KD < 0.1 nM) to calmodulin, CaM, a ubiquitous Ca^2+^-dependent regulator in eukaryotic cells. CaM binding triggers major structural re-arrangements in AC and stimulates its enzymatic activity more than a 1000-fold to reach the highest catalytic turnovers known for AC enzymes with a k_cat_ higher than 3000 s^−1^ in optimum conditions [43,44]. In the absence of CaM, AC still exhibits a residual activity of 1–3 s^−1^, which is on a par with many other AC enzymes (e.g., *E. coli* AC). Biochemical and structural studies revealed that the AC domain has a modular structure consisting of two subdomains, T25 and T18 [43,45]. The active site is located at the T25/T18 interface; hence, the isolated fragments are completely inactive. This observation led to the design of a biological screening tool for monitoring protein–protein interactions (PPI) in vivo in *E. coli*, the so-called bacterial adenylate cyclase-based two-hybrid (BACTH) system [46,47].

**Figure 3:**
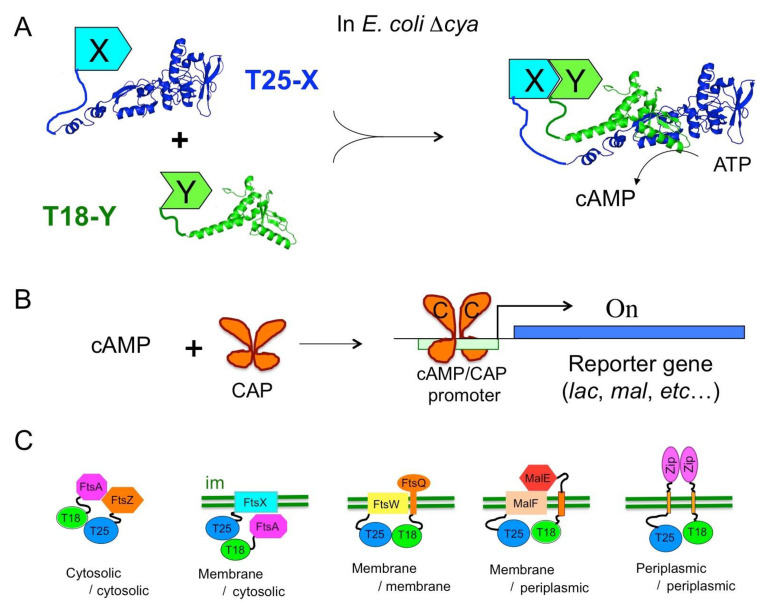
Principle of BACTH assay. (**A**) In an *E. coli cya* strain, the T25 and T18 fragments of CyaA are co-expressed as fusions with polypeptides X and Y; interaction between X and Y triggers heterodimerization of the hybrid proteins leading to cAMP synthesis. (**B**) The catabolite activator protein (CAP) binds cAMP and activates transcription of catabolic operons (reporter gene). (**C**) Diverse topological arrangement and subcellular localization of hybrid proteins; “im” represents the bacterial inner membrane.

Two-hybrid techniques are genetic assays based on the co-expression, in a reporter cell, of two hybrid proteins, which, upon interaction, produce a phenotypic or selective trait. Following the pioneering work of [48] who designed the yeast two-hybrid (YTH), many flavors of such systems have been elaborated using a variety of reporter proteins and in different organisms [49]. The BACTH system is based on functional complementation between the T25 and T18 fragments from CyaA (Figure 3). When the T25 and T18 fragments are expressed in an *E. coli cya* strain (i.e., lacking endogenous adenylate cyclase) as separate entities, they are unable to recognize each other and cannot reconstitute a functional enzyme. When the two fragments are fused to proteins that can associate (X and Y in Figure 3A), heterodimerization of these chimeric polypeptides results in functional complementation between the T25 and T18 moieties leading to the synthesis of cAMP.

This complementation can be easily tested in an *E. coli cya* strain (i.e., lacking endogenous adenylate cyclase), as cAMP is a pleiotropic regulator of gene expression in this bacterium. This nucleotide binds to the catabolite activator protein (CAP), a transcriptional activator that in turn binds to specific promoters and stimulates transcription of the many catabolite operons (Figure 3B). Therefore, bacteria expressing interacting hybrid proteins and producing cAMP are able to ferment carbohydrates such as lactose or maltose: they display a characteristic phenotype on indicator plates (e.g., Luria broth–X-gal or MacConkey/maltose) and can be selected on minimal media supplemented with lactose or maltose as unique carbon sources. Furthermore, the cAMP-induced expression of b-galactosidase can be easily monitored using many well-known colored (ONPG, X-gal) or fluorescent (MUG, FDG) substrates. Quantification of b-galactosidase expression in liquid cultures can be used to determine the relative efficiency of complementation [50,51,52]. Other *E. coli cya* reporter strains can be engineered to allow direct measurement of protein–protein interactions by luminescence or fluorescence by fusing appropriate reporters (e.g., GFP, lux operon) to a cAMP/CAP dependent promoter [53] (DL unpublished results). Other recently described implementations include adaptation of the T25 and T18 expression vectors to the Gateway recombineering technique to facilitate large scale analysis of interactions [54,55,56] and design of a BACTH *cya* strain that permits disulfide-bond formation in cytosol [57].

A major advantage of this two-hybrid assay results from the fact that it exploits a cAMP signaling cascade: consequently, the association of the two interacting proteins that triggers cAMP production can occur remotely from the transcriptional activation readout as cAMP is a diffusible messenger in the bacterial cell. Thus, the BACTH assay is capable of detecting interactions taking place in a variety of subcellular or topological locations. As illustrated in Figure 3C, in addition to interactions between cytosolic proteins—including proteins bound to DNA such as transcription regulators—it can report interactions between membrane proteins or between a membrane protein and a cytosolic one [52,58]. It can also probe interactions occurring in the periplasm (or between a periplasmic protein and an inner-membrane one) provided the periplasmic moiety is linked to the T25 or T18 fragments by a transmembrane segment (see Figure 3C) so that the cyclase fragments can remain in the cytosol [54]. Only proteins integrated into, or tightly associated with, the outer membrane of Gram-negative bacteria may be difficult to study with the current BACTH design.

Owing to its sensitivity and simplicity, the BACTH system has been widely exploited to characterize a wide variety of interactions between proteins from bacterial (the vast majority, of course), viral, mammalian or plant origins. Probing interactions of bacterial proteins with the BACTH system offers a major advantage as the corresponding proteins may be easily expressed in their native environment [52]. Indeed, the BACTH assay is particularly appropriate for studying the interactions implicated in multi-molecular, membrane-associated complexes from bacterial species. It has been instrumental in deciphering interactions involved in the assembly and dynamics of many machineries implicated in a wide variety of biological processes such as signaling, metabolism, cell division, secretion processes, transport systems, pathogenesis [59,60,61,62,63,64,65,66,67,68,69].

The BACTH system is a versatile assay that offers the possibility of both positive and negative selection. It can be easily applied to define the region(s) or domain(s) of a protein implicated in the association with a partner, and more precisely to identify the polypeptide residue(s) essential for the interaction. Many such examples can be found in the literature, and a few recent ones are given in references [70,71,72,73,74,75,76]. Such detailed analysis is important not only to better define the molecular basis of the protein–protein recognition but also to open the possibility of directly probing the physiological/functional significance of a given PPI by reverse genetics. In the above-mentioned bacterial machineries, BACTH has been instrumental in defining critical protein–protein interactions, the disruption of which were then shown to dramatically affect the corresponding physiological processes. This knowledge also paves the way to new therapeutic opportunities. Indeed, the development of inhibitors of protein–protein interactions (iPPI) able to prevent the formation of a protein complex represents a promising strategy for modulating fundamental biological processes [77,78].

The BACTH system can be easily adapted for high-throughput screening of PPI inhibitors as illustrated by Baron and colleagues, who used it for a large-scale screening of inhibitors of the bacterial type IV secretion system [79]. As noted above, the ability of the BACTH assay to monitor interactions between proteins expressed in their native environment (e.g., membrane) within live bacterial cells may facilitate the identification of small molecule inhibitors that have a better profile than those selected by classical in vitro assays. Indeed, a major drawback of such in vitro screens is that many identified molecules are ineffective in vivo because they cannot enter into bacteria. Further applications of BACTH assay in this field might help to develop novel antibacterial molecules urgently needed to cope with the emergence of bacterial antibiotic resistance.

Finally, we also recently implemented the architecture of the AC-based two-hybrid system to reach an extreme sensitivity. The standard design of the BACTH system relies on the “basal” enzymatic activity of AC obtained in the absence of CaM (k_cat_ ≈ 1 s^−1^) and a few hundred complexes of active hybrid proteins per cell are required to confer a selectable Cya+ phenotype in vivo. The newly implemented approach exploits the outstanding catalytic efficiency of AC upon stimulation by CaM (k_cat_ ≈ 2000 s^−1^) to drastically increase the sensitivity of detection. For this, a protein of interest is fused to CaM, while its potential partner is fused to an AC variant, ACm, modified by appropriate mutations to decrease its naturally very high affinity for CaM (Figure 3). Using appropriate mutation [19] and expression vectors fine-tuned to produce the lowest possible expression levels, this genetic assay was able to detect interactions with an extreme sensitivity corresponding to the detection of a single active complex per bacterial cell [80]. This highly sensitive system could be particularly appropriate for directing in vivo screening in bacteria of high affinity associations (e.g., with nanomolar affinities as both hybrid proteins are expressed at low nM levels) of recombinant single-chain antibody fragments or nanobodies [81,82] with their target antigens. It will also be useful for exploring the interaction network of many proteins that are expressed at low level in the cell and have been poorly studied until now.

## 4. Conclusions

The adenylate cyclase toxin presents several original properties, and some of them have been exploited for biotechnological and immunological purposes as described here. Other fundamental aspects of this essential toxin, like its calcium-dependent folding or its unique membrane translocation capacity, might be tamed as well in future innovative applications.

## Data Availability

Not applicable.
